# Facilitators and barriers to NCD prevention in Pakistanis–invincibility or inevitability: a qualitative research study

**DOI:** 10.1186/s13104-016-2087-2

**Published:** 2016-05-23

**Authors:** Ambreen Gowani, Hafiz Imtiaz Ahmed, Wardah Khalid, Abdul Muqeet, Saad Abdullah, Shariq Khoja, Ayeesha Kamran Kamal

**Affiliations:** Stroke Service Research Nurse, Aga Khan University, Karachi, Pakistan; Technology Development, Aga Khan Development Network, eHealth Resource Center, Karachi, Pakistan; Fogarty Cerebrovascular Research Fellow, The International Cerebrovascular Translational Clinical Research Training Program (Fogarty International Center, National Institutes of Health), Aga Khan University, Karachi, Pakistan; eHealth Innovation, Global, Aga Khan Development Network, eHealth Resource Center, Karachi, Pakistan; Tech4Life Enterprises, Karachi, Pakistan; Tech4Life Enterprises, Canada, and Technical Advisor-Evidence, Capacity and Policy mHealth Alliance, United Nations Foundation, Washington, USA; Section of Neurology, Department of Medicine, Stroke Fellowship Program, International Cerebrovascular Translational Clinical Research Training Program, Fogarty International Center and the National Institute of Neurologic Disorders and Stroke, Aga Khan University, Stadium Road, Karachi, 74800 Pakistan

**Keywords:** Non-communicable diseases, Self-management, Qualitative study, Lower and middle income countries

## Abstract

**Background:**

Non-communicable diseases (NCD) are the leading causes of death globally. In Pakistan, they are among the top ten causes of mortality, especially in the productive age group (30–69 years). Evidence suggests that health perceptions and beliefs strongly influence the health behavior of an individual. We performed focus group interviews to delineate the same so as to design the user interface of a non-invasive stroke risk monitoring device.

**Methods:**

It was a qualitative study, designed to explore how health perceptions and beliefs influence behavior for NCD prevention. Four focus group discussions (FGD) were conducted with 30 stable participants who had diabetes mellitus, ischemic heart disease, blood pressure, and stroke. The data was collected using a semi-structured interview guide designed to explore participants’ perceptions of their illnesses, self-management behaviors and factors affecting them. The interviews were transcribed and content analysis was done using steps of content analysis by Morse and Niehaus [10].

**Results:**

Medication adherence, self-monitoring of blood sugars and blood pressures, and medical help seeking were the commonly performed self-management behaviors by the participants. Personal experience of illness, familial inheritance of disease, education and fear of premature death when life responsibilities were unfulfilled, emerged as strong facilitators of self-management behaviors. A sense of personal invincibility, Fatalism or inevitability, lack of personal threat realization, limited knowledge, inadequate health education, health care and financial constraints appeared as key barriers to the self-management of chronic disease in participants.

**Conclusions:**

Behavioural interventional messaging will have to engender a sense of personal vulnerability and yet empower self-efficacy solutions at the individual level to deal with both invincibility and inevitability barriers to adoption of healthy behavior.

## Background

Non-communicable diseases (NCDs) like strokes and heart attacks are a major health issue worldwide and the mortality due to NCDs now exceeds that from communicable diseases [1–3]. Two thirds of NCD mortalities occur in low income developing countries which lack health literacy and resources [4]. Pakistan is no different where almost 25 % of all deaths are due to NCDs [5].

Prevention of NCDs requires sustained lifestyle changes. There is strong evidence indicating that individual perceptions and experiences of illness play an important role in their approach to disease preventive behavior. For instance, denial and lack of threat appreciation may result in non-adherence while perceived susceptibility may induce health- enhancing changes in an individual’s life [6–8]. Understanding the barriers and facilitators to adopting healthy habits versus deleterious ones are critical to designing successful interventions that would resonate with populations that are the targets of these behavior change programs.

For this paper, our definition of NCDs is illnesses which are linked by common modifiable risk factors such as; cardiovascular diseases, diabetes, hypertension, and stroke. In order to increase early recognition of modifiable risk factors that contribute to NCDs, our team is developing an all in one detection device which will be capable of detecting the participants’ 3- lead EKG, blood pressure, blood sugar and lipids non-invasively and provide health education messages that will enable early institution of NCD preventive behaviors based on these readings.

This study explores qualitatively the local perceptions on NCD and describes their self-management behaviors, facilitators and barriers to design and inform informational outputs that resonate with future interventions.

## Methods

### Study design

This is a descriptive exploratory study, using qualitative approach.

### Setting

The study was conducted at a tertiary care hospital in Karachi. The hospital is an internationally recognized tertiary care teaching hospital certified by Joint Commission International Accreditation (JCIA) that caters to the needs of large multi-ethnic urban population of 18 million. The annual outpatient volumes are about 600,000 a year, and inpatient volumes are 50,000 annually, with 577 + beds. There are in addition outreach programs within the community and outreach clinics and hospital. The services rendered encompass metabolic disorders, medicine, diabetes, cardiac care, and specialized stroke services and thus it was relatively easy to recruit and identify study participants.

### Sample and recruitment

A sample of approximately 16–20 participants was determined to explore the phenomenon of NCD prevention and self-care behaviors in our population. This sample was based on the concept of data saturation in qualitative design [9]. We increased the size of our focus groups until data saturation was achieved at 30 participants.

Participants were recruited from the out-patient clinics of the hospital which were cardiac, endocrine, stroke, general medicine, to best identify participants with NCD risk factors. Participants were invited for FGD based on eligibility criteria as follows: Age greater than 18 years, suffering from one of the NCD`S (diabetes, hypertension, coronary artery disease or stroke), attending AKUH clinics for their disease management and should be able to understand and communicate in Urdu. In order to ensure maximum variability among the sample, participants were purposively selected on the basis of their diagnosis, chronicity of the disease, age, gender, educational status, and type of the health facility being utilized (public or private).

### Data collection tool

A semi- structured interview guide was used to conduct the focus group discussion (FGD) (Table 1) The guide consisted of seven open ended questions that were designed to explore participants’ perceptions of their illnesses, self-management behaviors and factors affecting them.

**Table 1:** Qualitative interview guide

Interview guide for focus group interview
Did you ever know that you could suffer from this disease?
Probe: why did u think you could never get have this problem
Can you anticipate today that who in your family will acquire this disease in the future?
When do you check the status of your disease?
Probe: please elaborate
What problems do you encounter while going for testing?
How do you take care of your everyday issues related to your health problems?
Why do you think you take care of your health?
What prevents you from taking care of yourself?

### Ethical approval

This study was approved by the Ethical Review Committee Aga Khan University (ERC) Number 2891-Med-ERC-14.

### Study procedures

**Fig. 1:**
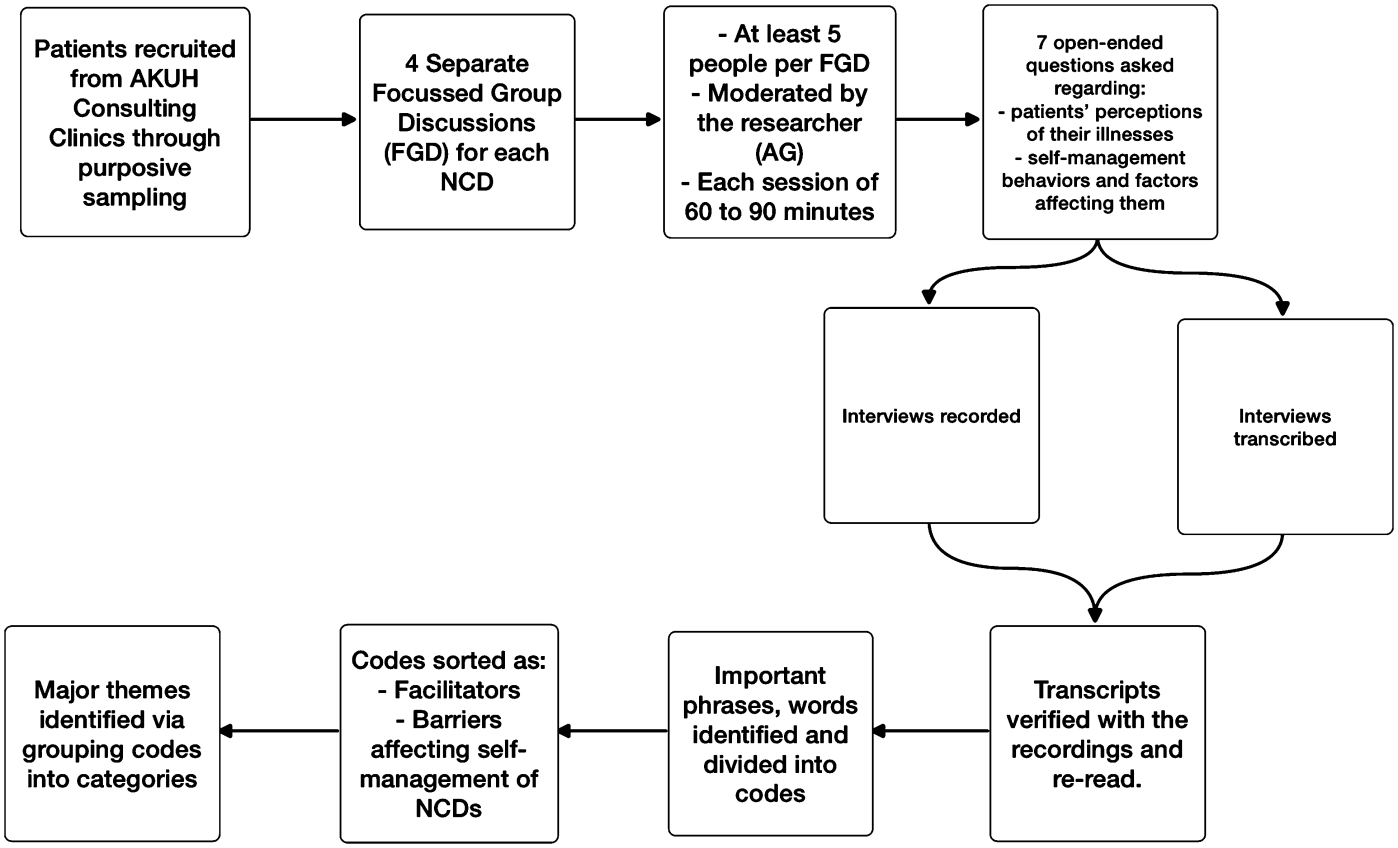
Qualitative study flow diagram. This figure illustrates the study processes

The participants were recruited by purposive sampling technique from all out-patient clinics. The participants were purposively selected from different clinics, based on their duration of disease, age, gender, socioeconomic and diverse ethnic background to ensure variability among study participants. A total of 30 participants participated in the FGD. The data collection continued till the saturation level was reached. In total four FGD’s were conducted from March till June 2014. For every above mentioned NCD’s a separate FDG’s was conducted, with at least five participants participating in each session. Each session lasted from 60 to 90 min and was moderated by the researcher (AG). The FGD’s were conducted at Clinical Trial Unit to ensure privacy of the participants. Each participant was counseled in detail regarding the study objectives and written informed consent was obtained. Interviews were only voice recorded and permission for recording was obtained from all participants. Strict privacy and confidentiality was maintained for all recordings and data. All interviews were performed in local language and transcribed within 7–10 days. The recordings were compared with the transcripts for verification to increase the accuracy of the data by the first author. The interviews were then translated to English (Fig. 1).

### Analysis

A thematic FGD’s guide was created by the experts in research team. The guide consisted of seven open ended questions that were designed to explore participants’ perceptions of illnesses, self-management behaviors and factors affecting them (Table 2). The included questions among others were: Did you ever know that you could suffer from this disease? When do you check the status of your disease? What features would you like to see in a device that can detect your blood physiology, non-invasively? How will you make the most of such device in managing your illness?

**Table 2:** Participants’ demographic and clinical characteristics

Participant’s characteristics	N (%)
Gender	
Male	17 (56.6)
Female	13 (43.3)
Age	
30–40	2 (6.6)
40–50	8 (26.6)
50–60	9 (30.0)
60–70	8 (26.6)
>70 years	3 (10.0)
Education	
Primary	11 (36.6)
Secondary	9 (30.0)
Graduate	10 (33.3)
NCD present	
CAD	10 (33.3)
Hypertension	20 (66.6)
Diabetes	15 (50.0)
Stroke	5 (16.6)
Duration of illness (years)	3 (1.5–10)^a^

^a^Median (IQR)

Qualitative manual content-analysis was performed to interpret the manifest content (what the text says) and the latent content (the interpreted meaning of the text) [10]. Following the steps of content analysis by Morse and Niehaus (5) [10] the transcriptions were read several times by the researchers to gain familiarity and understanding of the content. The interviews were transcribed and verified with the recording, by the researcher, to enhance the accuracy of the data. Content-analysis was used to interpret the data. Important words and phrases within the content were selected and the data was divided into meaningful units. After that the units were condensed and labeled with meaningful codes (either facilitators or barriers) affecting the self- management of NCD’s specifically hypertension, diabetes, stroke and coronary artery disease. First the coding was performed individually by two researchers and then consensus was reached on the final codes after discussion. The codes were further grouped together as sub-categories and then into categories. Then in the final step major themes were identified. The themes were collectively discussed and the final version of analysis was produced and agreed.

## Results

The results of the study are divided in two sections. The first part describes NCD preventive behaviors performed by the participants and the second part reports the factors that affect them. The key characteristics of the study participants are summarized in Table 2.

### Ncd preventive and self-management behaviors

**Fig. 2:**
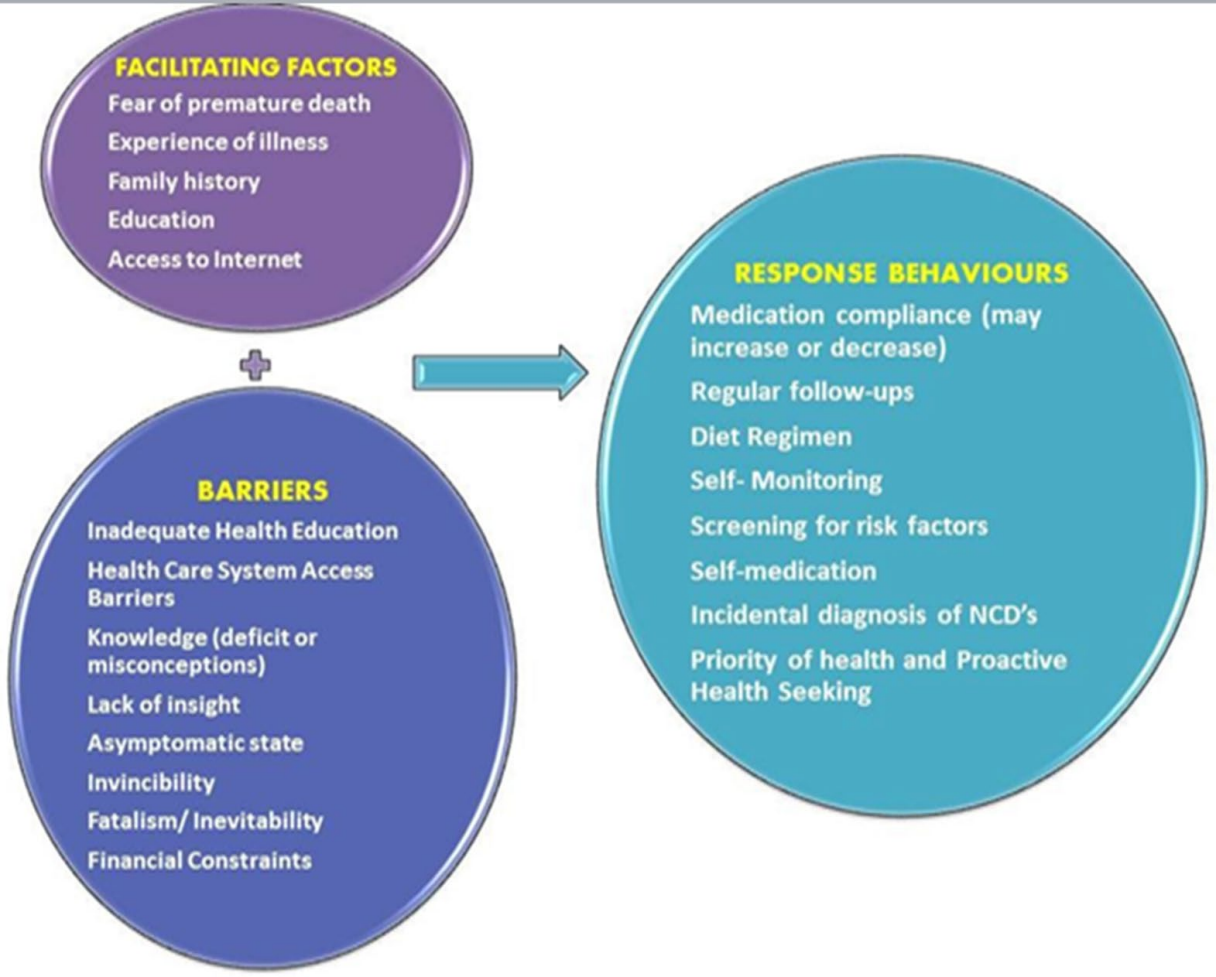
Conceptual framework of factors affecting NCD preventive behaviors. This figure illustrates the factors that motivate or block NCD preventive behavior

The most common self-management behaviors performed by the participants were adherence to medication regimen, regular exercise, medical help seeking and self-monitoring of blood pressure and blood sugar. They are discussed below in detail and expressed graphically in Fig. 2).

#### Medication adherence

Most of the participants reported higher adherence to medication regimen. They considered medications important for their wellbeing. The importance and adherence to medication was even greater among the participants who had experienced an adverse event because of non-compliance.

A 54 year old male said, *After my second angioplasty, I have become regular in taking medications because doctors told me that I got a heart attack because of stopping anti platelet agents by myself.*

In addition to that, participants preferred detailed instructions written in local language as it made their medication taking process simpler and easier. A 43 year old female shared,

*“My doctor writes everything clearly on my prescription, I just have to follow them”.*

#### Regular exercise

Regular exercise was not part of most of the participants’ lifestyle. Some did not consider it important for their health, whereas, others despite of being aware of its usefulness; were unable to incorporate them in their daily lives because of several reasons such as laziness, ignorance, lack of time, facility or presence of any other chronic diseases, such as Arthritis or vertigo.

A 65 year old woman shared her views on exercise as, *I have so much household work to do at home, that I don’t think I need to do exercise separately.*

#### Self-monitoring of blood pressure, glucose and cholesterol levels

Participants considered blood glucose, and blood pressure monitoring important to maintain their health status but did not worry much about their cholesterol levels. Even those who were on prescription medications, cholesterol levels remained unchecked. Most monitored their blood pressure and glucose because any change in their levels was believed by them to be “felt symptomatically” thus this monitoring was sporadic.

#### Adherence to diet

Another important behavior highlighted by the participants of the study was adherence to a dietary regimen. Females complied more strictly to diet regimen as compared to males. They restricted not only themselves but also their family members from excessively oily, salty and sweet food. A 32 year old diabetic female expressed, *I do not cook oily food and I do not let my family eat junk food, as I know what does getting diabetes means.*

#### Medical help seeking

All the participants verbalized the importance of regular follow ups and wanted to remain in touch with their physicians. However due to the expensive and time consuming hospital visits, they could not afford frequent follow ups. Hence, most of them did not visit their physician unless they were very unwell.

### Factors affecting NCD preventive behaviours

#### Barriers to NCD preventive and self care behaviors

##### Lack of threat appreciation

The analysis of participants’ narrative revealed that most of the participants in the study were unaware of the risk they carried for a particular disease, unless they or any of their family members encountered the disease itself. Therefore, most of the participants’ diagnosis of a certain disease was often a result of either an acute medical event (such as MI, or Stroke) or they discovered their risk when they sought help for some other medical condition. For example, a 43 year old hypertensive lady said,

*“I had headaches for weeks, I went to the doctor near my house, he checked my blood pressure and told me that I had high blood pressure…. Since then I am taking medications to keep my blood pressure under controlled.”*

Similarly, A 54 year old male shared, *“I was the healthiest member of my family, I had never been to any doctor before. When I had heart attack, my angiography showed that my three arteries were extensively blocked. It was unexpected.”*

Participants perceived themselves to be invincible. Prior regular screening, self-monitoring and risk identification was absent in almost everybody’s case except those whose family members either suffered from heart attacks or strokes or they were health care professionals.

Similarly, most of the participants sought medical help only when symptoms appeared. Hence, their illness remained undiagnosed for years unless it affected their functional status. Despite acknowledging their familial risk of an illness, its effects and complications, participants did not follow them seriously because of an inherent sense of invulnerability. However, after diagnosis, most of them tried making efforts towards healthy living. A 44 year old male shared,

“*I had never thought of getting MI, I had always remained healthy.“That day, I suddenly felt chest pain and I came to ER, I was rushed for angiography and angioplasty. It all happened just at that time… now I walk, check my sugar and take medications regularly.”*

#### Fatalism

Most of the participants strongly believed that getting a disease was their fate. It was meant to happen. It was unpreventable, and also unpredictable. They believed that even after prior identification of the risk factors nothing could not have stopped the occurrence of a stroke or heart attack. Those participants although, they followed their physician’s recommendations, still believed that disease progression would occur even after taking precautions. A 44 year old, male expressed his feelings as,

*“…. Whatever you do (to prevent the disease), what is written in your fate, it will happen eventually”.*

Participants who had strong perceptions of fatalism performed self-management casually as compared to those who believed that the effects of the disease could be minimized or delayed by following recommended lifestyle modifications.

#### Health care resource constraints

In addition to personal beliefs, participants highlighted limitations of the health care system such as unavailability and lack of communication, longer duration of follow-ups, and time consuming hospital visits. Consequently, these participants could not seek proactive medical help as they felt the system was inaccessible. A 48 year old diabetic woman shared,

*“It takes at least five hours to see the doctor here. One entire day gets ruined; I also have to take off from the job. Therefore, I only come to hospital when I feel unwell”.*

#### Knowledge deficit

Knowledge deficit regarding the illness, its parameters, and its management appeared common among almost all the participants which greatly influenced their self-management regimen. Participants could monitor their blood pressures and sugars but could not interpret them. Consequently they could not manage it themselves without any medical help. A 48 year old female with hypertension, shared,

*“I can operate the device and check my blood pressure, but I cannot tell whether it is high or low, unless somebody tells me”.*

#### Inadequate health education

Another important factor which emerged from the interview was insufficient health education. Lack of clarity, specificity and comprehensiveness in the health education affected participants’ self- management regimen. Physicians had told their participants about their diagnosis, prescribed them medications, but did not teach them self-monitoring and management of their illness. A 60 year old male with hypertension shared,

*“15* *years back, my physician told me that I had blood pressure. He did not tell me whether it was high or low. He gave me medications that I have been taking since then. I have just now come to know what is high blood pressure and what is low blood pressure”.*

#### Finance

Financial constraints were highlighted as the biggest barrier to self-management behaviors. High costs of physicians’ fee, diagnostic tests and cost of transportation compelled many to postpone their required health care needs. On the contrary, participants who could afford the cost, or had free access to medical services, had frequent follow ups and diagnostic checks.

A 61 year old female expressed her concern as: *It costs thousands of rupees only for the tests, and then you have to pay for doctor as well. I only get the tests when I feel something is wrong.*

### Facilitators to NCD preventive and self care behaviors

#### Experience of illness

Chronic disease participants were more aware and concerned about their illness as compared to the participants who were newly diagnosed. The past experience of serious events, hospitalization, financial burden and painful memories obliged them to engage in health-enhancing activities. The longer the participants had lived with their disease, the better was their knowledge and disease management. They were able to recognize their symptoms at an early stage perform self-monitoring and manage it through self-adjustment of medications. A 58 year old male shared,

*“I have high blood pressure since 15* *years, now I can measure my BP and manage it properly.”*

#### Education level of the patient

Educated participants had better understanding of their disease process as compared to their uneducated or less educated counterparts. Being able to read and write helped them add to their existing knowledge. They could communicate confidently with their physicians about their disease process. However, those who were uneducated could also perform self-management, but for them, it was a learning process while going through the experience of illness. Hence, being educated helped participants enhance their knowledge from sources other than their physicians, but apparently the self-management appeared similar in both educated and uneducated ones. A 36 year old educated lady with diabetes said,

*“I read somewhere symptoms of diabetes, so when I felt frequent micturition, I got a blood sugar test. And I was diagnosed having diabetes”.*

#### Familial inheritance of the disease

Participants, who have had any other family member suffering from any NCD, had greater awareness of the disease and its management. They also had an insight that they were more likely to encounter that disease. Hence, it shortened their denial phase and helped them accept the reality which eventually enhanced its self-management. A 55 year old male shared,

*“My father was diabetic, when I used to go with him for checkups; doctor told me that sooner or later you will also get diabetes. So I stopped taking sugar in tea and watched my diet. Now I am diagnosed with diabetes.”*

#### Fear of premature death

Another important factor that emerged from the narratives was fear of premature death due to which participants took care of themselves. They had an understanding that by keeping the levels under controlled, the early death could be prevented. A 50 year old lady expressed here fear as,

*“I take care of my diet, exercise, medications and check my sugar levels before every meal…. I don’t want to die early. I want to live for my children.”*

## Discussion

We assessed the barriers and facilitators to NCD prevention in Pakistanis using an open ended qualitative study design of focus group interviews. Our qualitative study revealed that most participants felt either no personal vulnerability to NCD, felt that they couldn’t do anything to change their outcomes and once they became victims they accepted their “fate”, without being active in changing their behavior (6) [7, 11, 12]. Those who had first-hand experience of illness in self or a relative had greater motivation to practice healthy behaviors to prevent the development of a potentially harmful NCD. In addition, those who were relatively better educated used social media to do something to adopt healthier lifestyles, another facilitator was the sense of responsibility and family (Table 3).

**Table 3:** Qualitative themes and sub-categories

Major themes	Categories	Sub-categories	Excerpts from the patients’ narratives
Factors affecting NCD preventive behaviours	*Positive factors/facilitators* Fear of premature health Experience of illness Familial inheritance of diseases Education level of the patient	*Contributing actions* Medication compliance Follow-ups/checkups Diet Regimen Self-monitoring	*“I never miss my medications, they are most important to me”* *“My father had diabetes; I knew I will get it, so I had already controlled my intake of sugar”*
	*Negative factors/barriers* Inadequate health education Health care system constraints Knowledge deficit Lack of insight about seriousness of disease No symptoms = no risk Invincibility/lack of threat appreciation Unpredictability of disease Fatalism Finance Cost of fresh food, vegetables and unsaturated oil	*Contributing actions* No regular screening Cost of the diagnostic tests Inability to interpret numeric values Self-medication Cholesterol screening not considered risk NCD diagnosed while seeking help for other medical conditions Sudden onset of acute events NCD are unpreventable Proactive help-seeking not a priority	*“10* *years ago, at the time of diagnosis, I did not know how much blood pressure was high, and how much was low. I learnt it over time, when I went through its fluctuating levels “* *“I stopped my medications after angioplasty for 2* *years…had another heart attack and had a By*- *pass then”* *“I check BP and sugar regularly because alterations in it make me nonfunctional”*

Although, we did not ask patients direct questions regarding their “*stage of change*” to actually change behavioral practices [13], these stages emerged from the discussion. Most patients were in the precontemplation or contemplation phase, and very few were actually practicing preventive lifestyle changes. Those who were motivated but due to knowledge deficit their motivation cannot be transferred into actions due to lack of support of working through obstacles.

In some ways our findings are similar to those reported in previous studies where medication compliance appeared to be the highest reported adhered behavior. [14, 15] Medication adherence was taken relatively seriously by the participants as compared to any other self-management behavior. Participants give importance to a written prescription. They find it authentic, important and inevitable because they are answerable to their physicians on the subsequent visit. Likewise, in our study, although participants preferred low salt, low cholesterol diet and tried to follow them, lack of knowledge about food choices hindered their dietary management. Cost of fresh vegetables and unsaturated fat also compelled them to compromise their regimen. This finding differs from other observations, where temptation for fast food and tastelessness were the major obstacles to diet regimen. [16–18].

Moreover, most of the participants in our study did not seek proactive medical help, missed their routine checkups and delayed their screening processes, thus, they ultimately presented to hospitals with acute catastrophic events like strokes and heart attacks. In the Pakistani context, 78 % pay out of pocket for health care and health insurance is a rare feature, spending on proactive medical help may not be a priority. [19] Furthermore, our participants felt that the health care system also hindered any participants’ initiative towards self-management such as, lack of communication between physician and patient, absence of support programs and telephonic help lines. Therefore, there is need to develop patient friendly self-enabling support systems which may perhaps utilize IT in the way that we intend to do.

We feel that we have used the open framework of a qualitative design and uncovered regionally important factors that we would not have done otherwise. Obtaining data on perceived sensitive factors faced by our participants demanded thorough understanding and planning of the content. The thematic guide was formulated after detailed discussions and consensus of local and global health experts. The researchers had thorough knowledge, were expert in local language and traditional meaning of content. Credibility was achieved by selection of context and well-structured questions. Transferability was achieved by purposeful selection of participants with diverse characteristics like gender, age, educational level, diverse cultural and ethnic background,different socioeconomic groups and participants suffering from four major NCD`s. Dependability was achieved by conducting interviews within 3 months to make sure that the phenomena under study did not change with time trends. Conformability was achieved through discussion on codes, sub-categories, categories and themes by the experts in research team. The conceptual frameworks of the Health Belief Model, Social Cognitive Theory, and Stages of Change informed the qualitative design (6, 7).

This study is limited in that we have limited our sphere of discussion to the community only. Similar open ended designs that elaborate the system key stakeholder perspectives may be useful future directions of research. However, it does clarify that any behavioral intervention to work in our context will have to engender a sense of vulnerability and yet empower self-efficacy at the individual level to deal with both invincibility and inevitability. It also elaborates broader challenges out of the scope of this project like health care systems reform, food policy changes and accessibility and equity within LMIC settings to prevent and mitigate the challenge of NCD [20–26].

## Conclusions

Our qualitative methodology clarified that besides the usual barriers to the practice and adoption of healthy lifestyle behaviors such as education and finance, the personal belief that one is either invincible; or that once an event happens, it was inevitable, will have to be targeted in counseling and public outreach messages to engender vulnerability in the first instance and self-confidence and efficacy in the second.

## Abbreviations

EKG: electrocardiogram
NCD: non communicable diseases
FGD: focus group discussion
AKUH: Aga Khan University Hospital
ERC: Ethical Review Committee
ER: emergency department
BP: blood pressure
IT: information technology
MI: myocardial infarction
LMIC: low and middle income countries
